# Phenotypic analysis of Longya-10 × pale flax hybrid progeny and identification of candidate genes regulating prostrate/erect growth in flax plants

**DOI:** 10.3389/fpls.2022.1044415

**Published:** 2022-12-06

**Authors:** Yanni Qi, Limin Wang, Wenjuan Li, Yaping Xie, Wei Zhao, Zhao Dang, Wen Li, Lirong Zhao, Jianping Zhang

**Affiliations:** Institute of Crop Sciences Gansu Academy of Agricultural Sciences, Lanzhou, China

**Keywords:** flax, wide hybridization, phenotypic analysis, BSA, growth habit

## Abstract

Flax is a dual-purpose crop that is important for oil and fiber production. The growth habit is one of the crucial targets of selection during flax domestication. Wild hybridization between cultivated flax and wild flax can produce superior germplasms for flax breeding and facilitate the study of the genetic mechanism underlying agronomically important traits. In this study, we used pale flax, *Linum grandiflorum*, and *L. perenne* to pollinate Longya-10. Only pale flax interspecific hybrids were obtained, and the trait analysis of the F_1_ and F_2_ generations showed that the traits analyzed in this study exhibited disparate genetic characteristics. In the F_1_ generation, only one trait, i.e., the number of capsules per plant (140) showed significant heterosis, while the characteristics of other traits were closely associated with those of the parents or a decline in hybrid phenotypes. The traits of the F_2_ generation were widely separated, and the variation coefficient ranged from 9.96% to 146.15%. The quantitative trait locus underlying growth habit was preliminarily found to be situated on chromosome 2 through Bulked-segregant analysis sequencing. Then linkage mapping analysis was performed to fine-map *GH2.1* to a 23.5-kb interval containing 4 genes. Among them, *L.us.o.m.scaffold22.109* and *L.us.o.m.scaffold22.112* contained nonsynonymous SNPs with Δindex=1. Combined with the qRT-PCR results, the two genes might be possible candidate genes for *GH2.1*. This study will contribute to the development of important germplasms for flax breeding, which would facilitate the elucidation of the genetic mechanisms regulating the growth habit and development of an ideal architecture for the flax plant.

## 1 Introduction

Flax (*Linum usitatissimum*) is the only species that has agricultural value within the Linaceae family. It is an annual, self-pollinated, diploid crop (2n=2x=30) (Zohary, 1999). Flax is one of the first crops to be domesticated, and has proven to be a rich source of oil and fiber for humans for more than 8,000 years (van Zeist and Bakker-Heeres, 1975). Nowadays, flax is grown in more than 30 countries, and is widely used as food, feed, and industrial raw material. The cultivation of flax plants exhibiting high yield, high quality, and strong resistance to stresses under different growth conditions is the main goal of flax breeding. However, the existing cultivated flax crops belong to the same species and have a narrow genetic basis (Smýkal et al., 2011; Pali et al., 2015; Vikas and Nandan, 2016; Hoque et al., 2020). In addition, the genetic diversity of flax cultivars has been reduced, owing to the long-term cultivation, artificial domestication, and excessive utilization of a few germplasm materials. Hence, it is difficult to achieve significant breakthroughs in yield and quality using conventional hybridization techniques, which severely limits flax breeding (Diederichsen and Richards, 2003; Diederichsen and Fu, 2006; Zhang et al., 2020). Wild flax resources exhibit unexploited genetic variations and many excellent characteristics, such as early maturation, tolerance to salinity and alkalinity, tolerance to barrenness, and resistance to cold and drought conditions (Diederichsen and Hammmer, 1995; Melnikova et al., 2014; Soto-Cerda et al., 2014; Sveinsson et al., 2014). Therefore, the efficient use of these germplasms can broaden the genetic background of flax, which is beneficial for the breeding of superior flax varieties.

Wide hybridization has been successfully used for the novel germplasm development and genetic improvement of many crops. Hybrids were successfully generated by the combination of a direct cross between *Capsicum annuum* × *C. baccatum* and *in vitro* embryo rescue (ER) (Manzur et al., 2015). Interspecific hybrids were obtained *via* hybridization between Japanese gentians and wild species of *Gentiana*, *Camelina sativa*, and its wild relative *C. microcarpa* (Takamura et al., 2019; Tepfer et al., 2020). Several studies on the distant hybridization of flax plants have been reported in recent years. Wang et al. (2007) and Mi et al. (2008) reported on several interspecific hybridization techniques and described the first interspecific hybrids of flax plants. Cheng et al. (2015) used cultivated flax and *L. grandiflorum* to study interspecific hybridization and ER, and preliminarily established the ER system. Interestingly, however, as the wild progenitor of cultivated flax, pale flax (*L. bienne*) has not been used in flax breeding so far (Diederichsen, 2007).

During flax domestication, changes in several characteristics, such as the seed size, capsule dehiscence, and growth habit, were observed (Diederichsen and Hammmer, 1995; Fu, 2011; Zhang et al., 2020). Hence, wild hybridization between cultivated flax and wild flax would not only facilitate the creation of new germplasms for breeding purposes, but also produce populations with contrasting traits that could be considered as important resources for the elucidation of the genetic basis of traits and identification of the candidate genes associated with variations in traits. During crop domestication, growth habit is an initial selection target modified in a direction that is more beneficial for yield improvement (Huyghe, 1998; Jin et al., 2008; Tan et al., 2008). For most crop plants, such as chickpea (Ali et al., 2015), rice (Hu et al., 2018), and wheat (Marone et al., 2020), the transition from prostrate growth to erect growth is a critical process, due to the potential advantages associated with an increase in photosynthetic efficiency, planting density, and crop yield. Cultivated flax plants exhibit the erect growth habit, while the prostrate growth habit can be observed in its wild progenitor, *L. bienne*, which is a winter annual or perennial plant species with dehiscent capsules, narrow leaves, and numerous branches (Diederichsen and Hammmer, 1995). Therefore, the erect growth habit is thought to be a more desirable trait for flax domestication, while the prostrate growth habit is considered to be an adaptation to disturbed habitats in the wild ancestor. The molecular mechanisms underlying growth habits have been studied in many plants, such as chickpea (Ali et al., 2015), soybean (Liu et al., 2010), rice (Hu et al., 2018; Li Z et al., 2019), barley (Zhou et al., 2018), maize (Tian et al., 2019), and wheat (Marone et al., 2020), and a series of quantitative trait loci (QTLs) and genes related to the growth habit have been identified and characterized. However, no study has reported on the growth habit in flax.

Bulked segregant analysis sequencing (BSA-Seq) is a rapid and efficient approach that has been developed for the identification of major QTLs or genes associated with a given phenotype (Michelmore et al., 1991; Kurlovs et al., 2019). BSA-Seq has been successfully used for mapping agronomically important loci in many plants, such as those associated with the branching habit trait in peanut (Kayam et al., 2017), leafy head formation in Chinese cabbage (Li R et al., 2019), genes controlling cold tolerance, QTLs controlling basal resistance to blast disease in rice (Guo et al., 2020; Liang et al., 2020), candidate genes involved in the development of curd riceyness in cauliflower (Zhao et al., 2020), a major QTL for shoot branching in *Brassica rapa* (Li et al., 2020), and genes related to melon resistance in *Phytophthora capsici* (Wang et al., 2020). Marker-assisted selection (MAS) is also an economical and efficient method, which has been successfully used in crops (Chen et al., 2021; Zhang et al., 2022). However, the studies on genetic mechanism analysis of important agronomic traits in flax were much lagging behind those of other crops.

In this study, we studied the compatibility of different crosses between wild flax and cultivated flax, and investigated and analyzed the main agronomic characteristics of hybrid progenies obtained from the Longya-10 × pale flax combination. Longya-10 and pale flax exhibit completely distinct growth habit; thus, the segregating population obtained from Longya-10 × pale flax can be used to identify the candidate quantitative trait loci (QTLs) and genes associated with growth habit. BSA-Seq and linkage mapping analysis were performed to explore and fine-map the major QTL responsible for growth habit. Our results would not only provide valuable resources for flax breeding, but also lay the foundation for further understanding the genetic mechanisms related to plant architecture.

## 2 Results

### 2.1 Wide hybridization analysis

#### 2.1.1 Comparison of different crosses

Under conventional conditions, Longya-10 × pale flax exhibited the highest level of cross affinity, and the cross-fruit setting rate was 30%, while the fruit setting rate of Longya-10 × *L. grandiflorum* was only 2.8% (**Table 1**). However, the ovary of Longya-10 × *L. perenne* was not enlarged, and no hybrid capsule could be obtained. Only two capsules obtained from Longya-10 × pale flax had seeds, and the average seed number per capsule was 2.67. However, no seeds were obtained from Longya-10 × *L. grandiflorum*. For the Longya-10 × pale flax combination, after all the seeds were planted, two seedlings emerged, and the emergence rate was 25%. One plant was a false hybrid with the same characteristics as the female parent, and the other plant was a true hybrid. A total of 248 F_2_ generation seeds were obtained, and 126 plants survived after all the seeds were sown (named LP-1 to LP-126).

**Table 1:** Comparison of characteristics between different crosses.

Cross combination	No. of crossed flowers	No. of fruit-setting flowers	Percentage of fruit-setting/%	No. of seeds obtained	No. of seeds per fruit	Percentage of germination/%	The true hybrid rate/%
Longya-10×pale flax	10	3	30	8	2.67	25	50
Longya-10×*L. grandiflorum*	36	1	2.8	0	0	0	0
Longya-10×*L. Perenne*	17	0	0	0	0	0	0

#### 2.1.2 Trait analysis of progenies obtained from Longya-10 × pale flax

**Figure 1:**
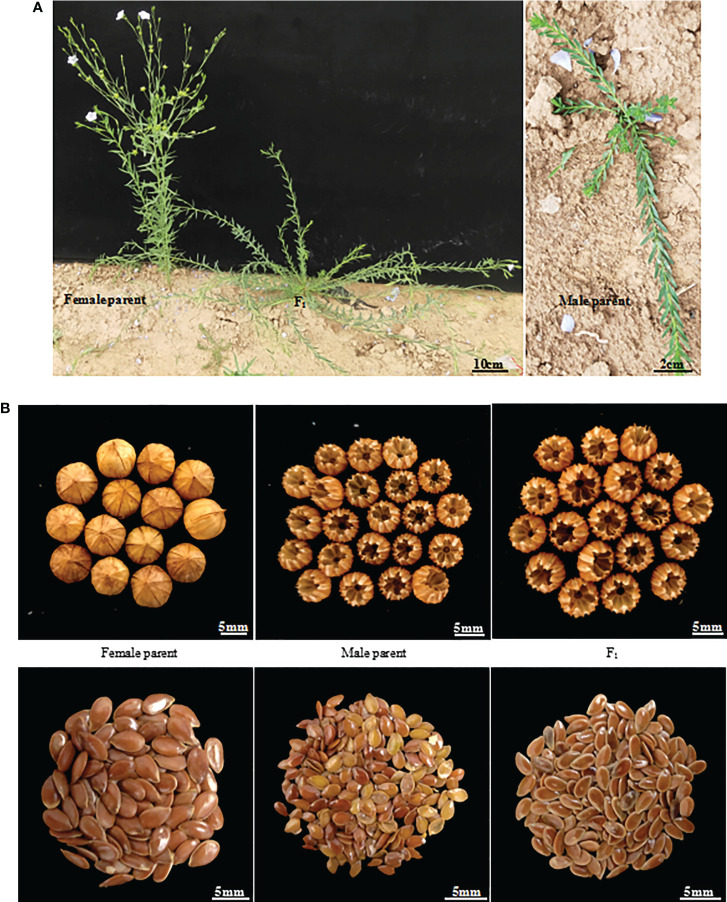
Morphological traits of parents and their generation line F_1_ of Longya-10×pale flax hybridization combination. **(A)** Growth habit; **(B)** Capsules and seeds.

Flowering was observed to occur in F_1_ generation plants only 7 days after it occurred in the female parent, Longya-10, but it was observed to occur at a much earlier stage than that for the male parent (**Figure 1A**). The growth period of the F_1_ plant was about 15 days longer than that of the female parent. A delayed flowering period was observed in the F_2_ generation, at about 70-95 days from planting to flowering, and approximately 10-35 days after that in the female parent. Among these plants, six plants (4.8%) grew very slowly, and died in winter without flowering. In thirty plants (23.8%), flowering was observed to occur 30 days after the occurrence of flowering in the female parent. In addition, the F_2_ generation had a longer growth period, and plants that could flower that year needed to be cultivated for about 130-160 days prior to the harvest period, which was 20-50 days more than that of the female parent. The growth cycle of different lines was remarkably different.

The growth habit of the F_1_ generation was observed to be of the semi-prostrate type without a main stem, and the number of branched stems was lower than the average number of branched stems in the parents (**Figure 1A**). The F_2_ generation had three types of growth habits, including the erect, prostrate, and semi-prostrate types (**Supplementary Figure 1**). Two plants grew upright and had obvious main stems. The growth habit of thirteen plants was of the prostrate type, of which one plant had no obvious main stem, and 12 other plants had no main stem. One hundred and eleven plants exhibited a semi-prostrate growth habit, of which 9 plants (8.1%) had no main stem, 11 plants (9.9%) had no obvious main stem, and the rest had an obvious main stem. The erect, semi-prostrate and prostrate growth habits of F_2_ generation exhibited a 1: 56: 7 segregation ratio, which characterized the trait of quantitative inheritance. The measurement results for the stem height (length) showed that there was a significant difference in the stem height among different lines (only the plants that bloomed that year were investigated), and the variation coefficient was 29.97 (**Table 2**). The plant heights of plants with main stems ranged from 21 to 97 cm, and the stem length of plants without main stems ranged from 30 to 107 cm. For plant height, the over mid-parent ratio was 56.67% (68 plants), and the over better parent ratio was 35.83% (43 plants). In addition, significant differences were observed in the number of branches among different lines; a variation coefficient of up to 75.59 was observed. The maximum number of branching stems was 20, while there were 30 plants without branching stems, accounting for 23.8% of the total number of plants.

**Table 2:** Single plant trait of hybrids obtained from Longya-10×pale flax.

Traits	Male parent	Female parent	Parents average	F_1_	F_2_							
					Maximum value	Minimum value	Range	Average	Over mid-parent/%	Over better parent/%	Standard deviation	Coefficient variation
Plant height(cm)	42.6	71.6	57.1	61.5	107	21	86	61.69	56.67	35.83	18.49	29.97
Stems per plant	72.4	1.8	37.1	27	20	1	19	4.63	0	0	3.50	75.59
Capsules per plant	56	64.4	60.2	140	181	1	180	32.07	16.30	15.22	38.42	119.80
Capsule diameter(mm)	4.72	7.29	6	6.32	6.58	4.22	2.36	5.32	5.43	0	0.53	9.96
Seeds per capsule	7.3	8.5	7.9	1.7	10	0	10	4.86	6.52	1.09	1.92	39.51
Seeds per plant	402.3	550.4	476.35	248	1147	2	1145	132.33	6.74	4.49	181.68	137.29
Thousand seed weight(g)	1.23	7.12	4.18	3.03	5.3	0.88	4.42	2.51	2.25	0	0.77	30.68
Grain weight per plant(g)	0.49	3.92	2	0.75	3.245	0.003	3.242	0.39	2.25	0	0.57	146.15

Capsules of the F_1_ generation were completely dehiscent. This was consistent with the capsules of the male parent, and the capsule size was slightly higher than that of the average capsule size of the parents (**Figure 1B**, **Table 2**). There were 92 plants from which capsules were harvested in the F_2_ generation, and capsule indehiscence was notably separated, including completely dehiscent, semi-dehiscent, and indehiscent capsules (**Supplementary Figure 2**). Among these, 10 plants were indehiscent (10.9%), 22 plants were completely dehiscent (23.09%), and the rest were semi-dehiscent, accounting for the majority of the F_2_ generation. The capsule size ranged from 4.22 to 6.58 mm, and the capsule sizes of 5 plants (5.43%) exceeded the mean capsule size of their parents. However, there was an absence of an over better parent plant. The variation coefficient of the capsule size was only 9.96.

The number of capsules per plant in the F_1_ generation was as high as 140, which was much higher than that of the better parent, while seeds per plant, thousand seed weight, and grain weight per plant values were lower than those of mid-parents (**Table 2**). The number of capsules per plant ranged from 1 to 181 in the F_2_ generation; significant differences were observed, and the coefficient of variation reached up to 119.30, of which the value in 15 (16.30%) individual plants exceeded the mean value of their parents, and that in 14 (15.22%) individual plants were superior to higher as compared to the values in their parents. In the F_2_ generation, the number of seeds per capsule ranged from 0 to 10, with a wide range of variations, and the coefficient of variation was up to 39.51. The seed number per plant ranged from 2 to 1147, and the variation coefficient was as high as 137.29. The thousand seed weight was significantly different, ranging from 0.88 to 5.3 g, and the variation coefficient was 30.68. The grain weight per plant varied widely (0.003-3.245 g), and the variation coefficient was 146.15.

#### 2.1.3 Genetic distribution of the F_2_ generation from Longya-10 × pale flax

**Figure 2:**
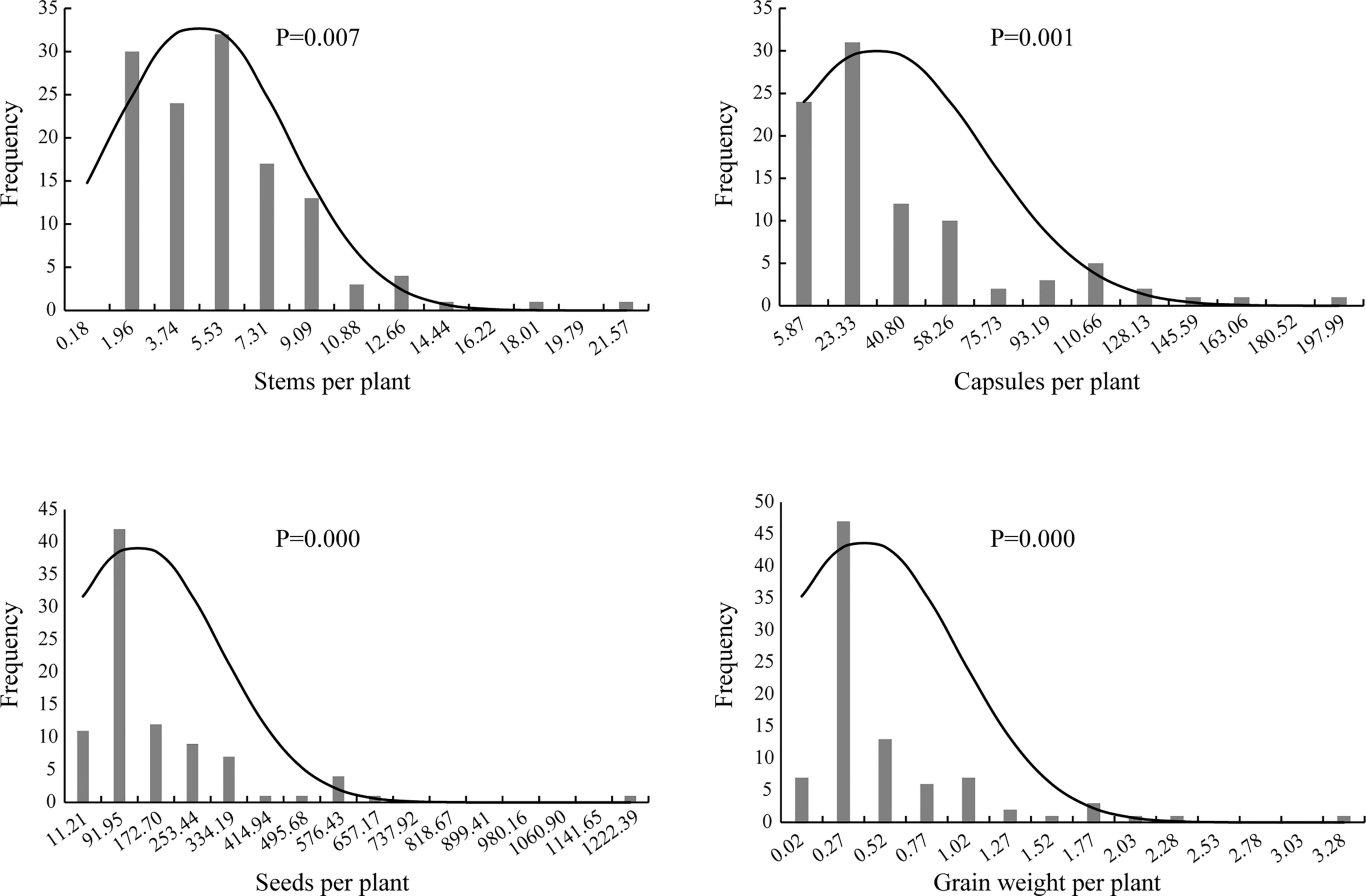
The histogram and normal distribution curve of traits per plant of F_2_ population of Longya-10×pale flax hybridization combination.

Genetic distribution analysis was conducted on the stem number per plant, capsule number per plant, seed number per plant, and grain weight per plant in the F_2_ generation (**Figure 2**). The stem number of individuals in the F_2_ generation was concentrated in three groups with the value of 1.96, 3.74 and 5.53, accounting for 68.25% of the total population, which indicated a skewed normal distribution (P <0.01). The capsule number per plant was concentrated in two groups on the far left, and accounted for 59.78% of the total number of plants; thus, a skewed normal distribution was observed (P <0.01). The seed number per plant was concentrated in one group with the value of 91.95, and accounted for 47.19% of the total population. The distribution frequency on the right side of this group was higher than that on the left side; thus, a skewed normal distribution (P <0.01) was observed. The grain weight per plant was concentrated in the second group on the left, accounting for 52.81% of the total. The number of individuals on the right side of this group was larger than that on the left, which exhibited a skewed normal distribution (P <0.01).

#### 2.1.4 Correlation analysis of agronomic traits of the F_2_ generation from Longya-10 × pale flax

The correlation analysis of the main agronomic traits of the F_2_ generation showed that the capsule size was positively correlated with the capsule number per plant, and the correlation was extremely significant (**Table 3**). The number of seeds per capsule was positively correlated with the capsule number per plant and capsule size, and the correlation was significant. The number of seeds per plant was positively correlated with the capsule number per plant and the number of seeds per capsule, with the correlation reaching significant and extremely significant levels, respectively. There was a significantly positive correlation between the thousand seed weight and capsule size. The grain weight per plant was positively correlated with the seed number per capsule, seed number per plant, and the thousand seed weight, among which the correlation with the seed number per capsule and seed number per plant was extremely significant, while the correlation with the thousand seed weight was significant. The correlation between the grain weight per plant and seed number per plant was the highest, with the correlation coefficient reaching 0.962.

**Table 3:** Correlation coefficient among agronomic traits of F_2_ population from Longya-10×pale flax.

Traits	Plant height	Stems per plant	Capsules per plant	Capsule diameter	Seeds per capsule	Seeds per plant	Thousand seed weight	Grain weight per plant
Plant height	—							
Stems per plant	0.010	—						
Capsules per plant	0.096	0.129	—					
Capsule diameter	0.014	0.189	0.562^**^	—				
Seeds per capsule	0.026	0.165	0.236^*^	0.252^*^	—			
Seeds per plant	0.146	0.128	0.210^*^	0.185	0.439^**^	—		
Thousand seed weight	0.020	0.029	0.155	0.238^*^	0.013	0.185	—	
Grain weight per plant	0.110	0.096	0.202	0.188	0.402^**^	0.962**	0.236*	—

**indicates significant differences at P<0.01, *indicates significant differences at the 0.05 level.

### 2.2 Initial mapping of growth habit by bulked-segregant analysis

#### 2.2.1 Construction and sequencing of the prostrate growth (PG) and erect growth (EG) bulk samples

In order to explore candidate regions and find genes associated with the growth habit of flax plants, PG and EG bulks were constructed for BSA-Seq analysis. After removing the low-quality reads, a total of 44,725,823 and 30,603,246 clean reads were obtained from the PG and EG pools, respectively. More than 90% of the reads were mapped to the reference genome, resulting in a coverage of over 90% with a depth of at least 10× in the two pools. Moreover, the average depth was over 29.73×, and the alignment efficiency reached at least 98.91% (**Supplementary Table 1**). We identified 1,862,228 and 605,255 SNPs, between Longya-10 and pale flax, and the EG-pool and PG-pool, respectively; among these, 72,282 and 21,826 were non-synonymous SNPs, respectively (**Supplementary Table 2**). A total of 304,291 and 111,397 insertions/deletions (InDels), including 4,391 and 1,526 frameshift InDels, were identified between Longya-10 and pale flax, and the EG-pool and PG-pool, respectively (**Supplementary Table 3**).

#### 2.2.2 Association analysis of BSA-Seq data

**Figure 3:**
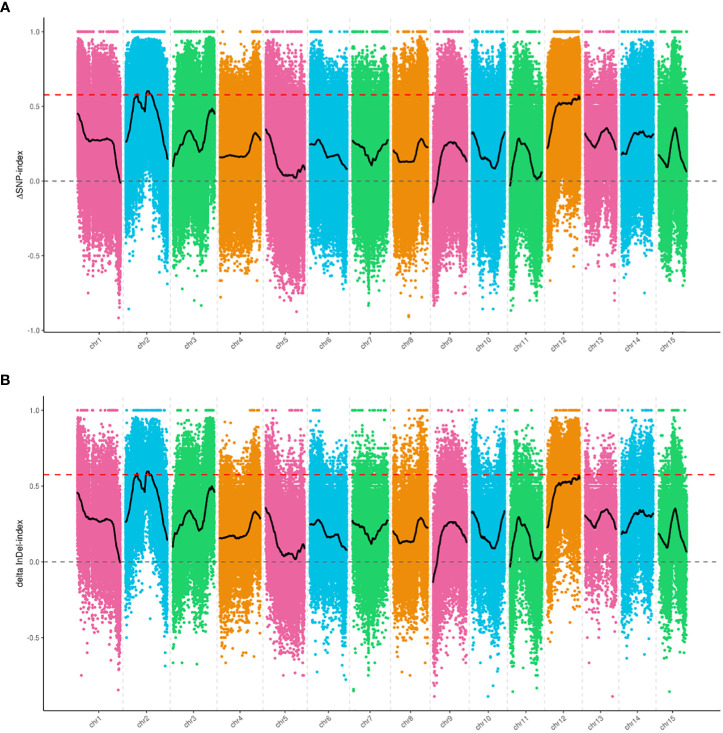
Candidate genomic regions for growth habit identified using **(A)** Δ(SNP-index) analysis and **(B)** Δ(InDel-index) analysis. The *X*-axis indicates the chromosome position, and the *Y*-axis indicates the Δindex values. The different colored points show the Δindex values calculated for specific SNPs or InDels screened on different chromosomes. The black curves are the fitted Δindex values. The red dashed lines represent the 99% threshold line.

We selected 1,465,819 SNPs and 235,561 small InDels that exhibited high quality for association analysis. The association analysis method involving Δ(SNP-index) and Δ(InDel-index) was used to identify the genomic regions responsible for the growth habit. Δ(SNP-index) analysis was used to identify two regions (a total length of 2.8 Mb), including 165 genes that were significantly associated with the growth habit (**Figure 3A**, **Table 4**). These regions were all located on chromosome 2. Using the Δ(InDel-index) method, four candidate regions containing 187 genes (a total length of 2.78 Mb) were identified (**Figure 3B**, **Table 4**), and all the regions were found to be situated on chromosome 2. Upon combining the candidate regions obtained using these two methods, the total length of the candidate regions, which included 209 genes, was found to be 3.16 Mb. The regions associated with growth habits were determined *via* an intersection of the candidate regions identified by the two methods. The total length of the region was 2.42 Mb and it contained 143 genes, out of which 103 genes were annotated (**Supplementary Table 4**). Four associated regions with a size of 0.15 Mb (from 6,000,000 to 6,150,000 bp), 1.88 Mb (from 10,750,000 to 12,630,000 bp), 0.29 Mb (from 12,890,000 to 13,180,000 bp), and 0.1 Mb (from 13,210,000 to 13,310,000 bp), which contained 18, 106, 15, and 4 genes, respectively, were identified.

**Table 4:** Candidate genomic regions identified by association analysis of BSA-Seq.

Association analysis method	Chromosome	Start	End	Size (Mb)	Gene number
Δ(SNP-index)	chr2	6000000	6150000	0.15	18
	chr2	10660000	13310000	2.65	147
	Total			2.8	165
Δ(InDel-index)	chr2	5810000	6220000	0.41	59
	chr2	10750000	12630000	1.88	106
	chr2	12890000	13180000	0.29	15
	chr2	13210000	13410000	0.20	7
	Total			2.78	187
Both	Total			3.16	209
Intersection	Total			2.42	143

Gene Ontology (GO) enrichment analysis was performed to understand the functions of all the 143 genes identified using both Δ(SNP-index) and Δ(InDel-index). The results indicated that 81 out of 143 genes were annotated to 32 categories associated with the cellular component, molecular function and biological process. In the cellular component, most of the candidates were assigned to the cell, cell part, and organelle. However, the binding and catalytic activity in the molecular function and metabolic process, cellular process, and single-organism process were enriched in the biological process category (**Supplementary Figure 3**). KEGG enrichment analysis of the candidate genes revealed that 18 pathways, which contained 22 genes, were found to be enriched (**Supplementary Figure 4**). These results showed that the growth habit may be associated with various biological processes.

Through an analysis of variants in candidate regions, 174 and 121 SNPs with non-synonymous mutations, and 8 and 5 InDels with frameshift mutations were identified between Longya-10 and pale flax, and the EG-pool and PG-pool, respectively. These variants might be directly related to the growth habit of flax plants (**Supplementary Tables 5 and 6**). A total of 141 genes identified using both Δ(SNP-index) and Δ(InDel-index) contained non-synonymous SNPs or InDels in the coding region, or variants in the promoter region, out of which 64 genes contained variants in the promoter region alone (**Supplementary Table 7**). Among the genes with variants, two genes (*L.us.o.m.scaffold22.109* and *L.us.o.m.scaffold22.112*) and seven genes (*L.us.o.m.scaffold22.97*, *L.us.o.m.scaffold22.100*, *L.us.o.m.scaffold22.112*, *L.us.o.m.scaffold123.25*, *L.us.o.m.scaffold177.28*, *L.us.o.m.scaffold228.4*, and *L.us.o.m.scaffold181.7*) contained SNPs (Δindex=1) between the EG-pool and PG-pool in the coding and promoter regions, respectively.

#### 2.2.3 Validation of qRT-PCR for candidate genes associated with growth habit of flax

**Figure 4:**
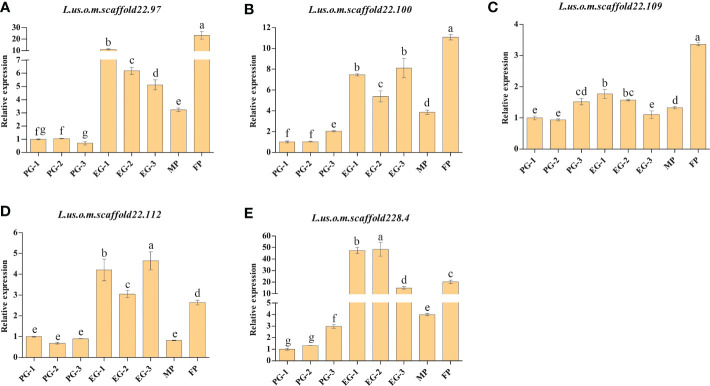
qRT-PCR analysis of the candidate genes in the parental plants and F_3_ generations. **(A)** *L.us.o.m.scaffold22.97*; **(B)** *L.us.o.m.scaffold22.100*; **(C)** *L.us.o.m.scaffold22.109*; **(D)** *L.us.o.m.scaffold22.112*; **(E)** *L.us.o.m.scaffold228.4*. MP: male parent; FP: female parent; PG: the basal section of stems from F_3_ generation with prostrate growth habit; EG: the basal section of stems from F_3_ generation with erect growth habit. The data are represented as mean ± SD of three biological replicates. The lowercase letters above the bars indicate the significant differences at the 0.05 probability level.

For the further validation of the eight genes containing SNPs with Δindex=1 between the EG-pool and PG-pool, qRT-PCR analysis was performed for Longya-10, pale flax, three F_3_ plants with prostrate growth habit, and three F_3_ plants with erect growth habit. According to the results, with the exception of *L.us.o.m.scaffold123.25*, *L.us.o.m.scaffold177.28*, and *L.us.o.m.scaffold181.7*, the expression levels of *L.us.o.m.scaffold22.97*, *L.us.o.m.scaffold22.100*, *L.us.o.m.scaffold22.112*, and *L.us.o.m.scaffold228.4* were significantly higher in Longya-10 and F_3_ with erect growth habit traits than in pale flax and F_3_ with prostrate growth habit traits (**Figure 4**). *L.us.o.m.scaffold22.109* was expressed at significantly higher levels in Longya-10 than in other individuals; however, the expression levels in F_3_ with erect growth habit traits were not always higher than those in plants with prostrate growth habit traits. This indicated that all the five genes might be candidate genes that facilitate the erect growth of flax.

### 2.3 Fine-mapping for growth habit in flax

**Figure 5:**
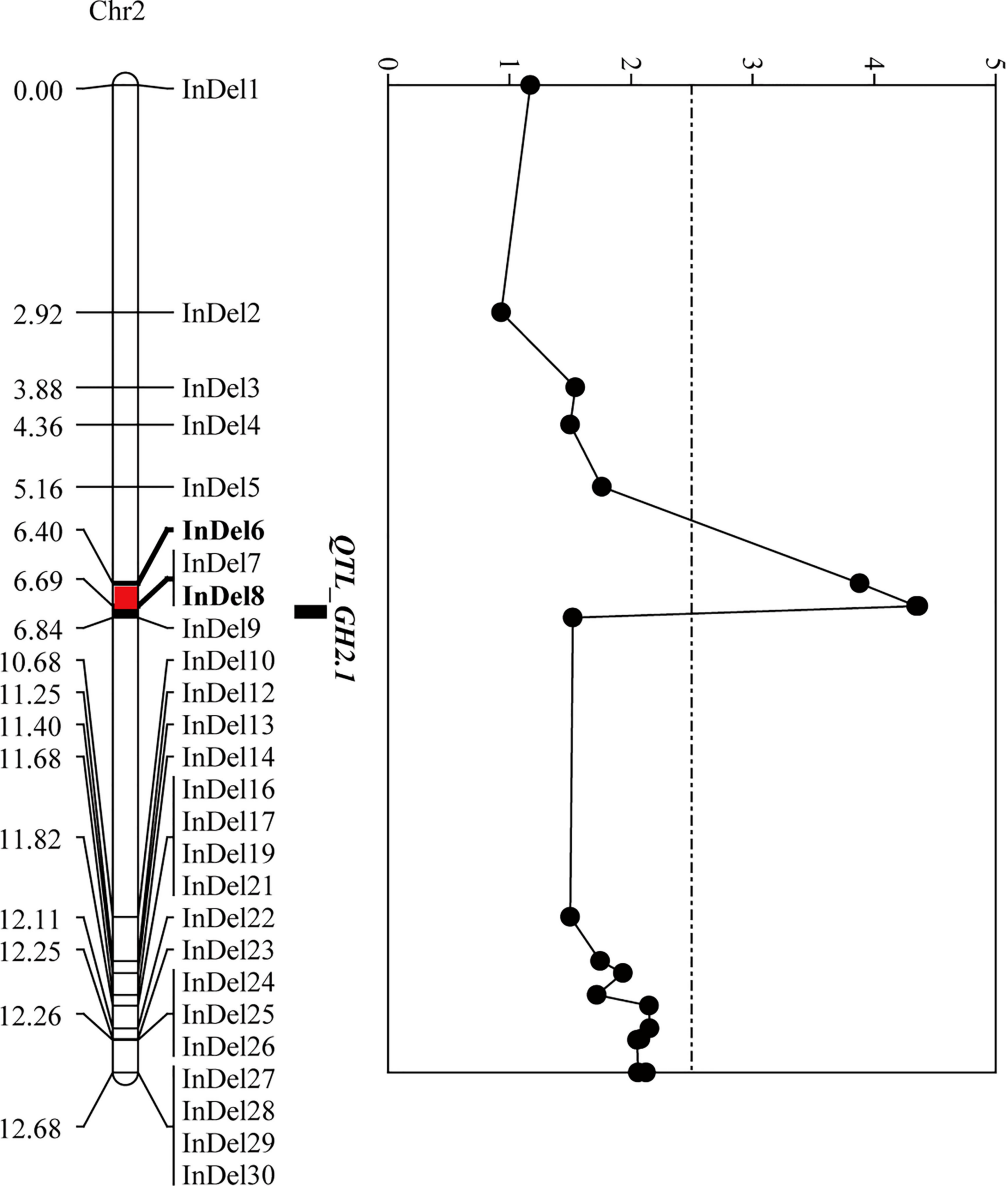
Fine-mapping of *GH2.1* by linkage analysis. The left panel exhibits the linkage map, in which the red segment indicates the positioning interval of *GH2.1*. The right panel shows the LOD value of each locus in F_2:3_ generation.

**Figure 6:**
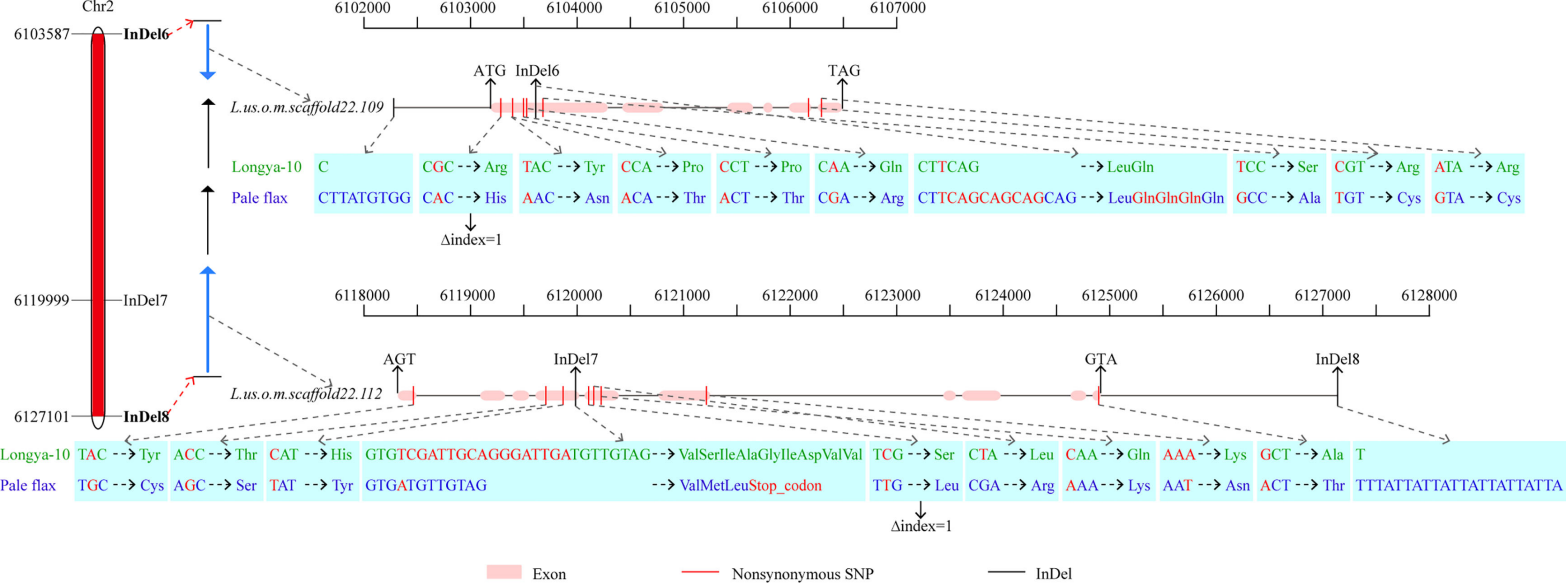
Fine-mapping interval of *GH2.1* and possible candidate genes within the region. The left bar shows the physical map of *GH2.1*. The middle arrow diagram display the genes in *GH2.1*, in which blue arrow indicate the genes *L.us.o.m.scaffold22.109* and *L.us.o.m.scaffold22.112*, which contained nonsynonymous SNPs with Δindex=1. The right section exhibits the variation sites of *L.us.o.m.scaffold22.109* and *L.us.o.m.scaffold22.112* between Longya-10 and pale flax.

To narrow down the regions and confirm the candidate genes obtained from BSA-seq analysis, a total of 31 InDel primers were developed, and used to detect polymorphism between the parents. Then 26 co-dominant primer pairs with polymorphism and clear bands were selected to identify the genotypes of 352 F_3_ individuals (**Supplementary Table 8**), and to construct a high-density genetic linkage map (**Figure 5**). Morever, QTL analysis was performed using the phenotypes of growth habit from F_2:3_ populations. The results showed that the region associated with growth habit was narrowed down between InDel6 and InDel8 with a physical distance of 23.5 kb, and named as *GH2.1* (**Figure 5**). *GH2.1* interval contained four genes, including *L.us.o.m.scaffold22.109*, *L.us.o.m.scaffold22.110*, *L.us.o.m.scaffold22.111* and *L.us.o.m.scaffold22.112*. All the four genes contained non-synonymous SNPs or InDels in the coding regions and variations (SNP and InDel) in the potential promoter regions, among which *L.us.o.m.scaffold22.109* and *L.us.o.m.scaffold22.112* contained both non-synonymous SNPs and InDels in the CDS regions (**Figure 6**; **Supplementary Table 7**). *L.us.o.m.scaffold22.109* contained eight non-synonymous SNPs and a three codon insertion (InDel6). *L.us.o.m.scaffold22.112* contained eight non-synonymous SNPs and a 16 bp deletion (InDel7), which could cause frameshift mutation and produced a stop codon; thus, the protein translation was terminated prematurely.

## 3 Discussion

### 3.1 Germplasm producing by wide hybridization

The genetic distance between wild flax and cultivated flax is much greater than that between cultivated flax. Moreover, wild flax has many beneficial characteristics. For example, *L. perenne* exhibits characteristics such as excellent disease resistance, many capsules per plant, drought resistance, and drought tolerance. *L. grandiflorum* has large red petals that are not found in cultivated flax and pale flax plants with more stems per plant (up to 72.4) and a perennial habit. Wide hybridization is an effective method that can be used for breeding new flax varieties. It can be used to introduce the beneficial variations of wild flax into cultivated flax, enrich the genetic background of cultivated flax, and generate potential heterosis. In addition, wide hybridization can produce important resources for the discovery of key related genes with important traits. As an ancestor of cultivated flax, pale flax exhibits beneficial alleles that could improve yield and unique alleles related to plant height and other important agronomic traits; hence, it can be used as the main germplasm for flax breeding (Diederichsen and Hammmer, 1995; Uysal et al., 2010; Soto-Cerda et al., 2014; Zhang et al., 2020). However, the hybridization of only *L. grandiflorum* and *L. perenne* with cultivated flax has been reported (Wang et al., 2007; Mi et al., 2008; Cheng et al., 2015). In this study, interspecific hybrids of Longya-10 × pale flax were successfully obtained under natural conditions; however, capsules without seeds were obtained from the Longya-10 × *L. grandiflorum* combination. No capsule was produced from the Longya-10 × *L. Perenne* combination. The cross affinity between pale flax and cultivated flax was the highest, followed by that of *L. grandiflorum* and *L. perenne*. This was attributable to the genetic relationship between the wild male parent and cultivated flax (Melnikova et al., 2014; Sveinsson et al., 2014; Zhang et al., 2020). In addition, pale flax has the same number of chromosomes as cultivated flax; hence, mature seeds can be obtained under natural conditions (Diederichsen, 2007).

### 3.2 Genetic characteristics of progenies obtained from Longya-10 × pale flax

It was found that the capsule indehiscence in the F_1_ generation was consistent with that of the male parent, indicating that the capsule dehiscence was controlled by the dominant gene. The genetic characteristics associated with the flowering time were in accordance with maternal preferences, while those associated with the grain weight per plant were in accordance with the paternal preferences. The capsule number per plant in the F_1_ generation was significantly higher than that observed for the parents, and showed a significant level of heterosis, which was attributable to the genetic heterogeneity of both the parents (Chen et al., 2009). There were significant differences in the molecular and phenotypic characteristics between the pale flax and cultivated flax plants (Diederichsen and Hammmer, 1995; Uysal et al., 2010; Zhang et al., 2020). The characteristics of growth habit, plant height, capsule size, thousand seed weight, and stem number were between the two parents. The seed number per plant and seed number per capsule were lower than the lowest values observed for the parent, which was indicative of the phenomenon of decline in hybridization levels. In this study, only one plant belonging to the F_1_ generation was obtained; hence, as the number of hybrids was too small, the genetic characteristics of each trait need to be studied further.

The characteristics of the F_2_ generation were widely isolated, and the coefficient of variation ranged from 9.96% to 146.15%. The coefficients of variation of the characteristics were in an order of grain weight per plant > seed number per plant > capsule number per plant > stem number per plant > seed number per capsule > 1000-grain weight > plant height > capsule size. In addition, three types of growth habit traits (prostrate growth, semi-prostrate growth, and erect growth) and capsules (dehiscent capsules, semi-dehiscent capsules, and indehiscent capsules) were observed in the F_2_ generation. In this study, the genetic variation coefficient of capsule size was the smallest with a mediocre selection potential. Higher genetic variations were observed in the yield-related traits and stem number; hence, the potential for genetic improvement was greater. After further selection, superior individuals meeting breeding goals could be selected from the offspring. In the present study, a high proportion of over mid-parent (56.67%) and over better-parent (35.83%) was observed only for the plant height trait; other characteristics were either not found or were associated with a very low proportion of over mid-parent and better-parent. This might be because of the small offspring population resulting from small combinations. There was a significant or extremely significant positive correlation between yield-related traits, but no significant correlation was observed with the plant height and stem number, which was different from those of previous studies (Chen et al., 2009; Wang et al., 2013). During the breeding selection process, the rational use of the correlation between important traits could improve the efficiency of the breeding process. The population in this study was small and all offspring were derived from one F_1_ plant; hence, the correlation between each of the traits needs to be discussed further.

### 3.3 Identification of major QTLs for growth habit using efficient strategy

The transition from prostrate growth (pale flax) to erect growth (cultivated flax) is very important during flax domestication. Detecting the major QTLs for growth habit will not be beneficial for understanding the underlying molecular mechanisms, but also be helpful to promote the molecular improvement of the architecture of the flax plant. BSA-Seq is a very popular method used to elucidate the genetic basis of agronomic traits in many species, because of the simplicity associated with the sample collection and data analysis processes (Kayam et al., 2017; Li R et al., 2019; Guo et al., 2020; Vogel et al., 2021) . In this study, four candidate regions were identified by BSA-Seq, which had a total size of 2.42 Mb and contained 143 genes. Five genes (*L.us.o.m.scaffold22.97*, *L.us.o.m.scaffold22.100*, *L.us.o.m.scaffold22.109*, *L.us.o.m.scaffold 22.112*, and *L.us.o.m.scaffold228.4*) with ΔSNP index equal to 1 were expressed at a significantly higher levels in plants growing upright. However, the candidate regions identified through BSA-seq are usually large and containing lots of genes, so the traditional mapping approach is still needed in validating and narrowing down the regions further (Zhang et al., 2022). In the current study, we constructed a genetic map to fine-map *GH2.1* to a shrinking 23.5-kb interval using an F_3_ population. The procedure applied in this study promoted the mapping of major QTLs and could be used in the identification of other QTLs in flax.

### 3.4 Functional annotation of possible candidate genes in *GH2.1*

In the confidence interval of *GH2.1*, two genes *L.us.o.m.scaffold22.109* and *L.us.o.m.scaffold22.112* contained non-synonymous SNPs with Δindex equal to 1, which also harbor other variations in both the coding and promoter regions. *L.us.o.m.scaffold22.109* is homologous with *URT1* (*UTP: RNA uridylyltransferase 1*) in *Arabidopsis*, and was found to participate in a molecular network connecting several translational repressors/decapping activators (Scheer et al., 2021). *L.us.o.m.scaffold22.112* encodes an SPX domain-containing membrane protein belonging to a major facilitator superfamily (SPX-MFS proteins) that act as a vacuolar Pi transporter. This transporter played a critical role in the regulation of cellular Pi homeostasis, which was required for fitness and plant growth (Liu et al., 2016). Luan reported that vacuolar Pi transporters controlled the fine-tuning of Pi allocation, which was particularly vital for reproductive development in *Arabidopsis* (Luan et al., 2019). Although the two genes are not homologous with the reported genes that could affect the plant growth habit, such as *PROG1* (Jin et al., 2008; Tan et al., 2008), *PROG7* (Hu et al., 2018), *RPAD* (Wu et al., 2018), *LAZY1* (Li et al., 2007; Dong et al., 2013), *TAC1* (Yu et al., 2007), *LPA1* (Wu et al., 2013), *TIG1* (Zhang et al., 2019) and *BRXL14* (Yoshihara and Spalding, 2020), in our study, they showed a markedly higher expression level in erect individuals than in prostrate individuals; hence, they were regarded as candidate genes whose specific function needed to be analyzed further.

## 4 Materials and methods

### 4.1 Plant materials and parental combination

Longya-10, which grow upright, was bred in our laboratory. Pale flax, *L. grandiflorum*, and *L. perenne* were obtained from certain geographical areas and allowed to grow in our laboratory, among which pale flax show a prostrate growth habit. Cross-breeding experiments were conducted at the Gansu Academy of Agricultural Sciences from 2017 to 2018. Because pale flax takes about ten months to bloom and cannot bloom in the winter, they were planted in a climate chamber in 2017, while *L. grandiflorum*, *L. perenne*, and Longya-10 were planted in a field in 2018. Interspecific hybridization was performed at the flowering stage. In the afternoon, we selected Longya-10 flower buds that would blossom the next day, removed their petals and anthers, and covered them with hybridization bags. Fresh pollen from *L. grandiflorum*, *L. perenne*, and pale flax was smeared on the stigmas of Longya-10 the next morning. The stigmas were immediately covered with hybridization bags, and hang tags were used to indicate cross combinations. We collected seeds from the hybridized plants at maturity.

In 2019, all the seeds obtained from Longya-10 × pale flax combination were sown in a field. Based on the morphological characteristics of F_1_ plants, we removed the false hybrids, and the true hybrids were self-fertilized to develop segregating population. In 2020, all seeds collected from the F_1_ plants were sown in the field, and each F_2_ plant was self-fertilized, then the seeds were collected individually from each plant. In 2021, the progenies of each F_2_ plant were grown in a line to develop the F_2:3_ population. One month after the planting process, the surviving F_3_ plants displayed three types of growth habits, including the erect, prostrate, and semi-prostrate types (**Supplementary Figure 1**). From these, 60 plants with contrasting growth habits, which were selected from different lines, were uesd to establish F_3_ segregation bulks (30 with prostrate growth habits and 30 with erect growth habits) for BSA-Seq analysis.

The female parent (Longya-10) and the male parent (pale flax) were sampled for real-time PCR analysis. Individual plants with highly distinct growth habits (3 with prostrate growth habits and 3 with erect growth habits) were also selected from the F_3_ generation. Samples were obtained from the base of the stems of all the plants. All samples were frozen in liquid nitrogen and then stored at -80°C for analysis.

### 4.2 Trait analysis

The phenotype of the F_1_ generation was studied, and the pseudohybrids with the same characteristics as the mother parent were eliminated. We examined the traits of the F_1_, F_2_, and F_3_ generations throughout the growth period. The traits assessed included the flowering stage, stems per plant, plant height, growth habit, and presence or absence of a prominent main stem. The fruit setting rate, capsular indehiscence, number of capsules per plant, number of seeds per plant, grain weight per plant, number of grains per capsule, capsule size, and thousand seed weight were investigated at maturity. SPSS 19.0 and Microsoft Excel were used to process and analyze the data for the investigated traits.

### 4.3 Bulked-segregant analysis methods

#### 4.3.1 Construction of an illumina library for BSA-Seq

For BSA-Seq, a total of 60 plants (30 with prostrate growth habit and 30 with erect growth habit) were selected from the F_3_ population for bulking. Two DNA pools were constructed by mixing equal amounts of genomic DNA from 30 individual plants with prostrate growth habits (PG-pool) and 30 individual plants with erect growth habits (EG-pool). The two sequencing libraries were prepared in accordance with the standard Illumina protocol and were then sequenced on an Illumina NovaSeq 6000 platform (Genepioneer Biotechnologies, Nanjing, China). The sequencing data of the two parental lines were obtained *via* the whole-genome sequencing of Longya-10 and pale flax (Zhang et al., 2020).

#### 4.3.2 Sequence alignment and variant calling

Fastp (v0.20.0) was used for the cleaning and filtering of reads. Low-quality reads containing adaptors, in which the proportion of N (failed to identify the specific base type) was greater than 10%, and more than 50% of bases with a Q-score that was lower than 10 were excluded, and clean reads from each pool were subsequently aligned to the flax reference genome sequence (https://www.ncbi.nlm.nih.gov/nuccore/QMEI00000000.2) using BWA software (Li and Durbin, 2009). SAMtools (v1.9) was used to convert the generated SAM files to the BAM format (Li et al., 2009). Duplicate reads were removed to mask the effects of PCR duplication by using the MarkDuplicates tool in Picard (http://sourceforge.net/projects/picard/), followed by local realignment and base recalibration using GATK software (McKenna et al., 2010). HaplotypeCaller and VariantFiltration in GATK were used to perform the variant calling of SNPs and small InDels, and for filtering low-quality variation sites; reliable variants were subsequently obtained (McKenna et al., 2010). Finally, the SnpEff software was used for the annotation of SNPs and small InDels (Cingolani et al., 2012).

#### 4.3.3 Mapping of candidate genomic regions by association analysis

To identify candidate regions associated with certain growth habits, SNPs/InDels with multiple genotypes, or with read support less than 4, or with consistent genotypes among the mixed pools, and the SNPs/InDels whose recessive pooled genes were not from recessive parents were all filtered out. The DISTANCE method was also used to fit the Δ(SNP-index) and Δ(InDel-index), and regions with a threshold value above 99% were selected as the candidate regions related to the growth habit using both methods (Takagi et al., 2013).

#### 4.3.4 Gene annotation in candidate regions

For the annotation and functional analysis of genes in the candidate regions, we used Blastall (v2.2.26) to compare the genes with functional databases, including the GO (Ashburner et al., 2000), COG (Tatusov et al., 2003), KEGG (Kanehisa et al., 2004), NR (Deng et al., 2006), SWISS-PROT (Deng et al., 2006), and other databases, to obtain the optimal results and functional information regarding genes, with an e-value of 10^-5^. The enrichment analysis of genes in associated regions was also conducted.

### 4.4 Real-time quantitative PCR for validation

Total RNA was extracted from Lonagya-10, pale flax, and F_3_ plants with different phenotypes (prostrate growth and erect growth) using the Plant Easy Spin RNA Miniprep Kit (BIOMIGA, USA). We assessed the RNA quality and quantity using a NanoDrop2000 spectrophotometer (Thermo Fisher Scientific, Wilmington, USA) and agarose gel electrophoresis. For each sample, 1 µg of total RNA was used for cDNA synthesis by using the PrimeScript™ RT Reagent Kit with gDNA Eraser (Perfect Real Time; TaKaRa), in accordance with the recommended protocol. cDNA used as the template for qRT-PCR was diluted with RNase-free water. The qRT-PCR was performed according to the method described by Zhang et al. (2020). *GAPDH* served as the reference gene for flax plants (Huis et al., 2010). The relative expression levels of candidate genes were calculated by using the 2^−ΔΔCT^ method (Antonov et al., 2005). The expression analysis of all samples was performed in triplicate. The specific primer pairs of candidate genes were designed using Primer Premier 5.0 (PREMIER Biosoft International, USA) and are listed in **Supplementary Table 9**.

### 4.5 Linkage map construction and fine-mapping

To narrow down the candidate regions identified by the BSA-seq analysis, InDels between Longya-10 and pale flax were selected from the candidate regions. InDel primers were designed according to the flanking sequences of the target InDels. First, the primers were used to screen the parents, then the primers with polymorphism were selected to detect the genotype of the F_3_ individuals. These primers are listed in **Supplementary Table 10**. The QTL IciMapping V4.1 software was used to construct the genetic linkage map, convert the recombination values into genetic distances (in centimorgan), and finally perform the fine-mapping analysis of the QTL in F_2:3_ populations (Meng et al., 2015). MapChart 2.2 was used to visualize the genetic map and QTL information (Voorrips, 2002).

## Data availability statement

The datasets presented in this study can be found in https://doi.org/10.6084/m9.figshare.13614311.v3. and DDBJ/ENA/GenBank under the accession QMEI02000000 and QMEG00000000.

## Author contributions

JZ conceived and designed the experiments. YQ, WenjL, LZ and WenL performed the experiments and data analyses. YQ analysed the data wrote the manuscript. LW and YX contributed to the experimental design and coordination of the study. WZ and ZD contributed to the interpretation of the results. All authors have read and agreed to the published version of the manuscript. All authors contributed to the article and approved the submitted version.
