# Supplementary material for: Phenotypic analysis of Longya-10 × pale flax hybrid progeny and identification of candidate genes regulating prostrate/erect growth in flax plants
Source: Front Plant Sci. 2022 Dec 6;13:1044415. doi: 10.3389/fpls.2022.1044415 (PMC9763623; doi:10.3389/fpls.2022.1044415)
Supplement: Supplementary file 1 [file DataSheet_1.pdf]

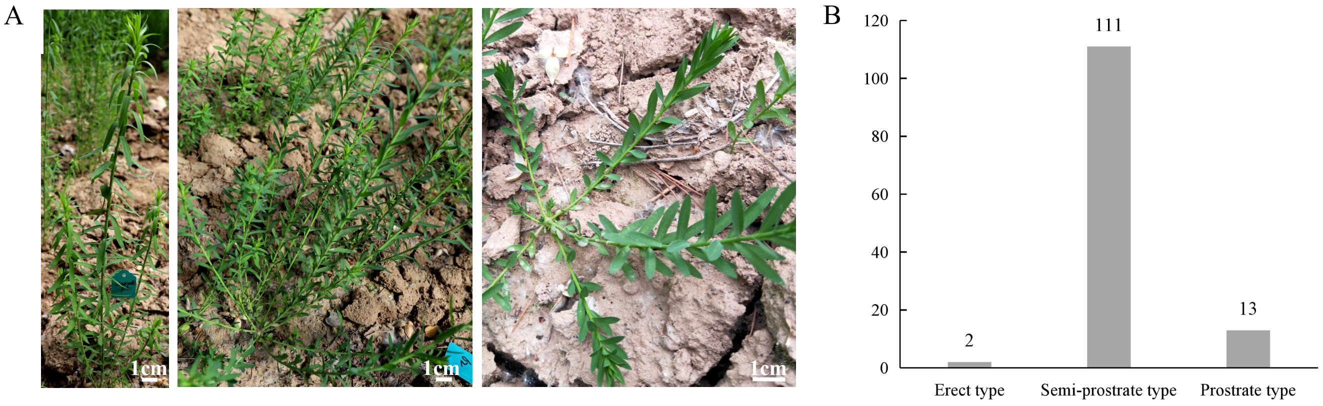

Supplementary Figure 1. Investigation on growth habit of F_2 population from Longya-10×pale flax hybridization combination. (**A**) Growth habit; (**B**) The distribution of each type of growth habit.

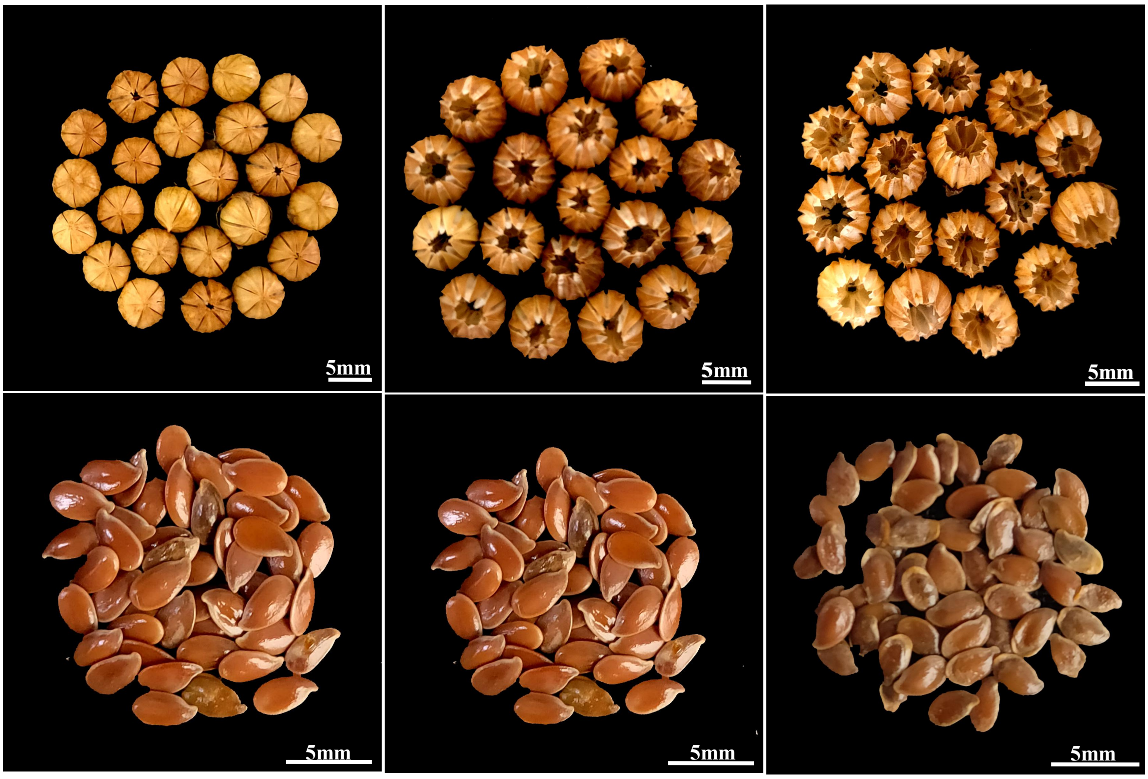

Supplementary Figure 2. Investigation on capsules and seeds of F_2 population from Longya-10×pale flax hybridization combination.

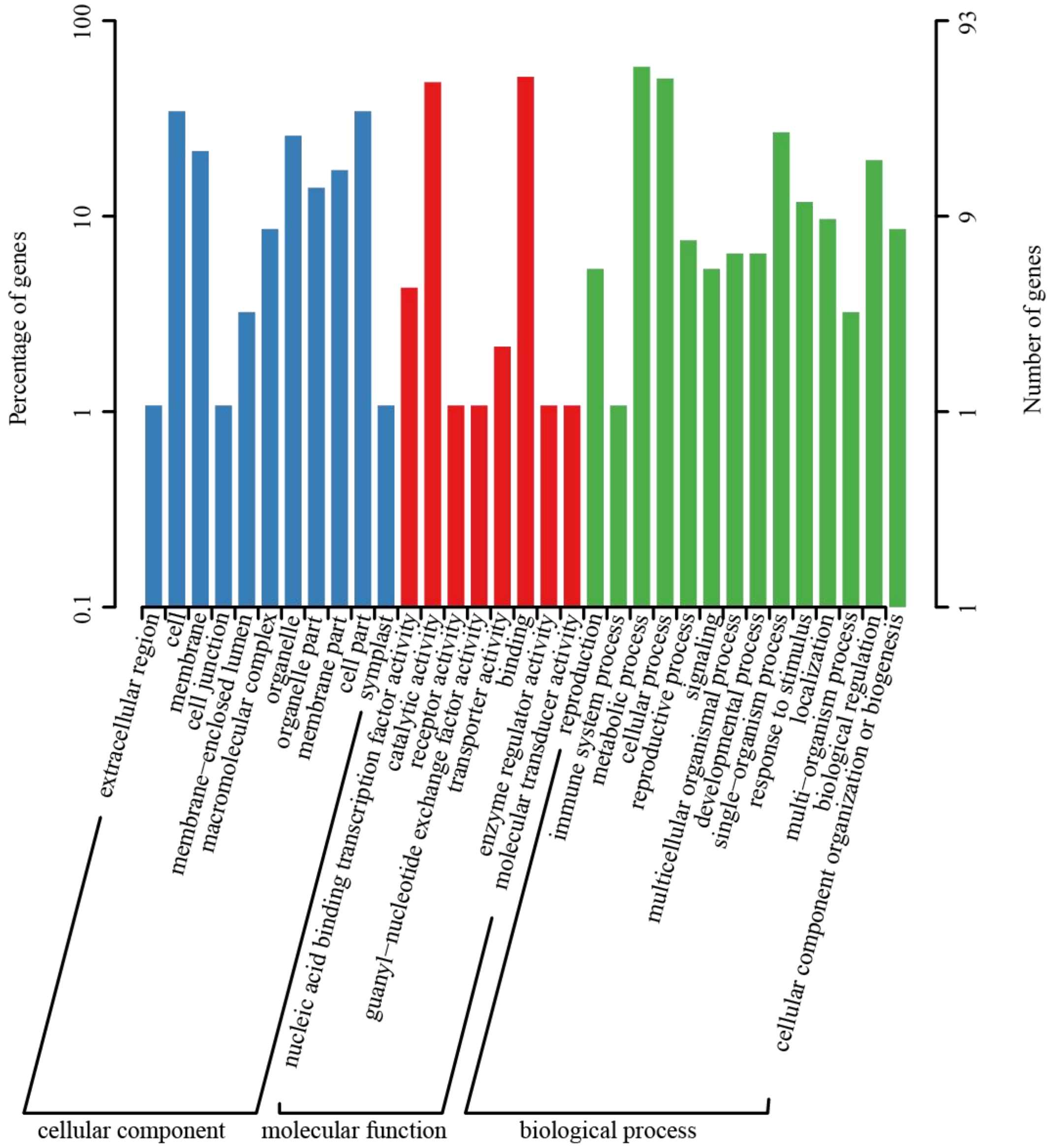

Supplementary Figure 3. GO enrichment analysis of candidate genes selected by both Δ(SNP-index) analysis and Δ(InDel-index) analysis.

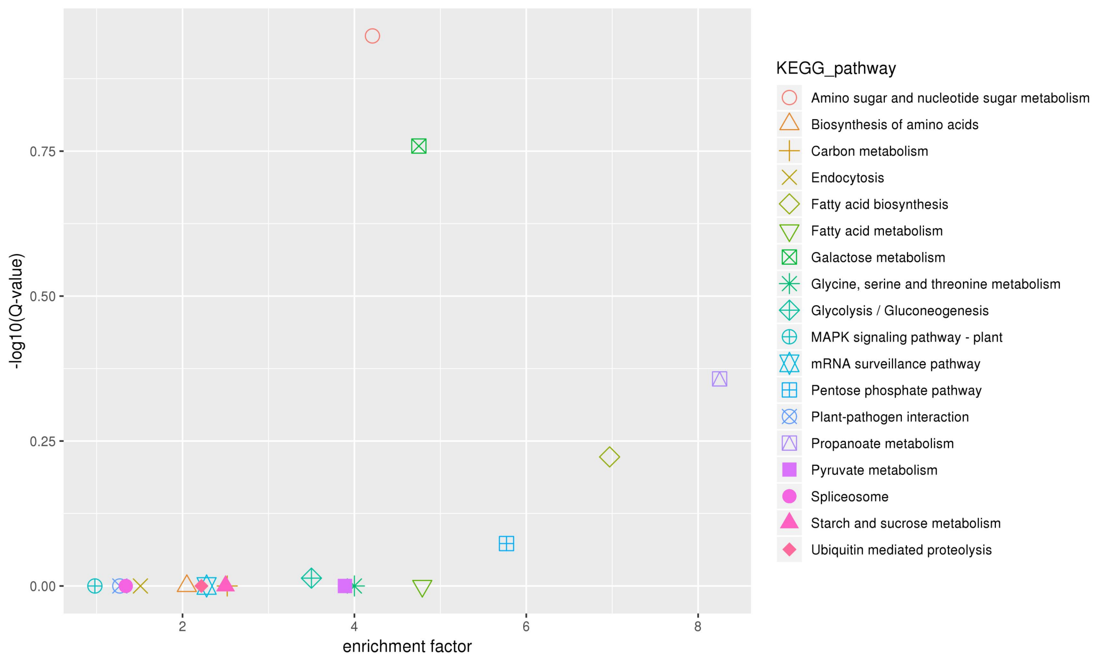

Supplementary Figure 4. KEGG pathway enrichment analysis of candidate genes selected by both Δ(SNP-index) analysis and Δ(InDel-index) analysis.
